# Respiratory Microbiota Associations with Asthma Across American and Emirati Adults: A Comparative Analysis

**DOI:** 10.3390/applmicrobiol5030059

**Published:** 2025-06-29

**Authors:** Ariangela J. Kozik, Kyra Henderson, Laila Salameh, Bassam Mahboub, Mohammad T. Al Bataineh, Yvonne J. Huang

**Affiliations:** 1 Department of Molecular, Cellular, and Developmental Biology, College of Literature, Science, and the Arts, University of Michigan, Ann Arbor, MI 48109, USA; 2 Rashid Hospital, Dubai Health, Dubai P.O. Box 4545, United Arab Emirates; 3 College of Medicine, Yarmouk University, Aydoun P.O. Box 566, Jordan; 4 Center for Biotechnology, Khalifa University, Abu Dhabi P.O. Box 127788, United Arab Emirates; 5 School of Medicine and Health Sciences, George Washington University, Washington, DC 20052, USA; 6 Department of Medicine, Division of Pulmonary/Critical Care Medicine, University of Michigan, Ann Arbor, MI 48109, USA; 7 Department of Microbiology/Immunology, University of Michigan, Ann Arbor, MI 48109, USA

**Keywords:** airway, microbiome, asthma, geography

## Abstract

**Background:**

Clinical features of asthma are associated with differences in the lower airway microbiome. However, knowledge is limited on whether airway microbiota composition differs between individuals residing in different geographic regions and if asthma-associated differences in lower airway microbiota are similar between distinct populations.

**Methods:**

Existing 16S rRNA gene sequence data, generated from sputum collected from adults with or without asthma (*n* = 74) from two single-center cohort studies in the U.S. and United Arab Emirates, were re-processed for merged computational analysis using standard available tools. Potential differences between study sites, asthma status and specific clinical factors (inhaled corticosteroid use, ICS; obesity) were examined.

**Results:**

Differences in sputum bacterial composition, assessed by alpha- and beta-diversity measures, were associated with study site. Despite this, asthma-related differences were discerned in both cohorts. Specifically, sputum microbiota of asthmatic patients on ICS treatment displayed reduced bacterial phylogenetic diversity, compared to those not on ICS treatment (*p* = 0.006). Sputum bacterial composition also differed by obesity status (unweighted Unifrac distance PERMANOVA, *p* = 0.004). Specific genera were identified in both cohorts that were differentially enriched between obese vs. non-obese subjects, including *Rothia* and *Veillonella* (obesity-associated) and *Campylobacter* (non-obesity-associated).

**Conclusions:**

Our findings suggest clinical factors associated with differences in the lower airway microbiome in asthma may transcend variation related to geographic area of residence.

## 1. Introduction

Asthma remains highly prevalent worldwide. While clinical outcomes across different populations have been extensively studied [1], aspects of lower airway biology have less often been directly compared, particularly between patients from distinct geographic regions. In addition to well-studied inflammatory pathways in asthma [2], recent studies of adults with asthma have reported asthma-associated differences in the airway microbiome, including altered microbiota compositions related to asthma phenotype and outcomes. For example, differences in the lower airway microbiome have been associated with degree of type 2 airway inflammation, asthma severity and control, and even obesity-associated asthma [3-10].

Research interest in the lower airway microbiome has grown exponentially in the last decade, yet published data on asthma and other chronic airway diseases has largely come from patients residing in highly studied countries and regions around the world. A question not uncommonly asked of investigators in this field is whether there are major differences in the composition of lower airway microbiota between individuals living in different geographic regions and especially different climates. Within the U.S., studies conducted by NIH-supported research consortia that have focused on asthma or COPD have not found location of clinical centers to be a significant factor in explaining variation in airway microbiome composition, in contrast to disease status and other clinical variables [4,5,7,11,12]. However, this finding is restricted to data from patients residing in or around major U.S. cities or population areas with generally temperate climates. Thus, one open question is whether there are major differences in the lower airway microbiome between individuals, with or without asthma, living in temperate versus arid climates for example. The effects of environmental differences, including climate factors, on chronic lung disease have been well documented [13-15]. However, limited data exist on whether differences in climate, or specific exposures related to climate, are associated with changes in the respiratory microbiome. Moreover, such data on the lower airway microbiome in humans (versus upper airway/nasal microbiome) is particularly sparse [16-19]. Lastly, whether asthma-related clinical factors that have been associated with differences in the lower airway microbiome [3,4,8-10] are similar between patients residing in climactically different locales is also unknown. We hypothesized that lower airway microbiota composition differs by geographic region, and that asthma-related airway microbial differences are conserved across different geographic regions.

To further explore this, we performed a pilot study analyzing previously generated sputum microbiota data from adults residing in the U.S. (Southeast Michigan, USA) [8] and the United Arab Emirates (Sharjah, U.A.E.) [20]. The prevalence of asthma in Emirati adults has been reported to be 10–15% [21], with sub-optimal asthma control not uncommon [21-24]. A prior study [20] reported differences in sputum bacterial and fungal microbiota composition between Emirati adults with or without asthma, an observation broadly concordant with findings from other adult asthma cohorts [3,4,24]. While the sputum microbial data were already generated, we note that sputum collection unavoidably includes a mix of lower and upper airway secretions but is much less invasive and often preferred clinically by patients and by research participants (compared to performing more invasive bronchoscopy, for example). Analysis of sputum has been relied upon extensively in the study of chronic airway diseases, including asthma, and provided insights into both microbiome and inflammatory features linked to differences in disease outcomes or phenotype [2-4,25-28]. Using the existing sequence data, our main goal in this study was to compare the composition of sputum bacterial microbiota between American and Emirati adults and explore whether any microbiota features related to asthma were similar or different between the groups.

## 2. Materials and Methods

This study re-analyzed previously generated 16S rRNA gene sequence data from sputum samples collected from U.S. and Emirati adults with and without asthma in previously described studies [8,20]. The Emirati dataset came from a case–control study where investigators collected spontaneous expectorated sputum samples from Emirati citizens with and without asthma. For that study, individuals were excluded if they reported use of antibiotics and/or were prescribed probiotics within the past 3 months, reported any form of smoking, or had other respiratory diseases or infections. The U.S. dataset came from a prospective observational study of adults with and without asthma. The exclusion criteria for this study included a significant history of smoking, recent asthma exacerbation, reported use of antibiotics within 8 weeks prior to enrollment, or reported immunosuppressive therapy. Specifically, for the U.S. patients (*n* = 55), 16S rRNA gene sequencing data was generated from asthmatic and healthy adults enrolled in a single-center observational study (NCT02887911; clinicaltrials.gov) at the University of Michigan, Ann Arbor. For the adult patients from U.A.E (*n* = 19), existing 16S rRNA gene sequencing data were derived from sputum collected at University Teaching Hospital, Sharjah, U.A.E., as previously described (approved by the Dubai Scientific Research Ethical Committee DSREC-10/2020-58) [20]. 16S rRNA gene sequencing targeting the hypervariable V4 region was used in both studies.

All participants were non-smokers, and all asthmatic adults included in this study had mild or moderate asthma severity according to GINA criteria [29]. Asthma diagnosis was confirmed in the parent clinical studies [8,20] by spirometry. The median asthma control test score was slightly lower in the U.A.E. group (18 ± 3 (standard deviation) vs. 21 ± 4) (Table 1).

In both parent studies [8,20], 16S rRNA gene sequencing of sputum DNA was performed on the same platform (Illumina MiSeq, Illumina Inc. San Diego, CA, USA) using the same dual-barcoded primers targeting the V4 region and following standard Illumina sequencing protocols. The raw 16S rRNA sequence files (.fastq) from both cohorts were re-processed together in QIIME2 (version 2019.7) [30], and amplicon sequence variants (ASVs) determined using DADA2 [31] and matched to taxonomies using the SILVA database [32,33]. This yielded a merged dataset with identically processed sequence data from both cohorts. Bacterial taxa comprising <0.1% of the total community were removed, and ASV counts were converted to relative abundance where appropriate for downstream analyses. For alpha-diversity analyses (within-sample diversity), read number was rarefied to 1266 to allow inclusion of all samples at the same depth level to calculate measures of community richness, evenness, Shannon diversity, and phylogenetic diversity (Faith index) [34]. Beta-diversity analyses (between-sample variation) were performed using either Bray–Curtis or unweighted and weighted Unifrac distances [35] in QIIME2.

The following variables available from both U.S. and U.A.E. cohorts were examined for associations with airway bacterial microbiota composition: study site, sex, patient type (asthma vs. no asthma/healthy control), treatment with inhaled steroids, and presence of obesity (defined by body mass index ≥ 30 kg/m^2^). The MicrobiomeAnalyst platform [36] was used to perform taxonomic level analyses for differentially abundant organisms, including by linear discriminant analysis effect size (LEfSe) [37] and edgeR [38]. Non-parametric tests (Kruskal–Wallis) were used where appropriate for other between-group comparisons.

## 3. Results

### 3.1. Sputum Bacterial Community Composition by Study Site

**Figure 1.**
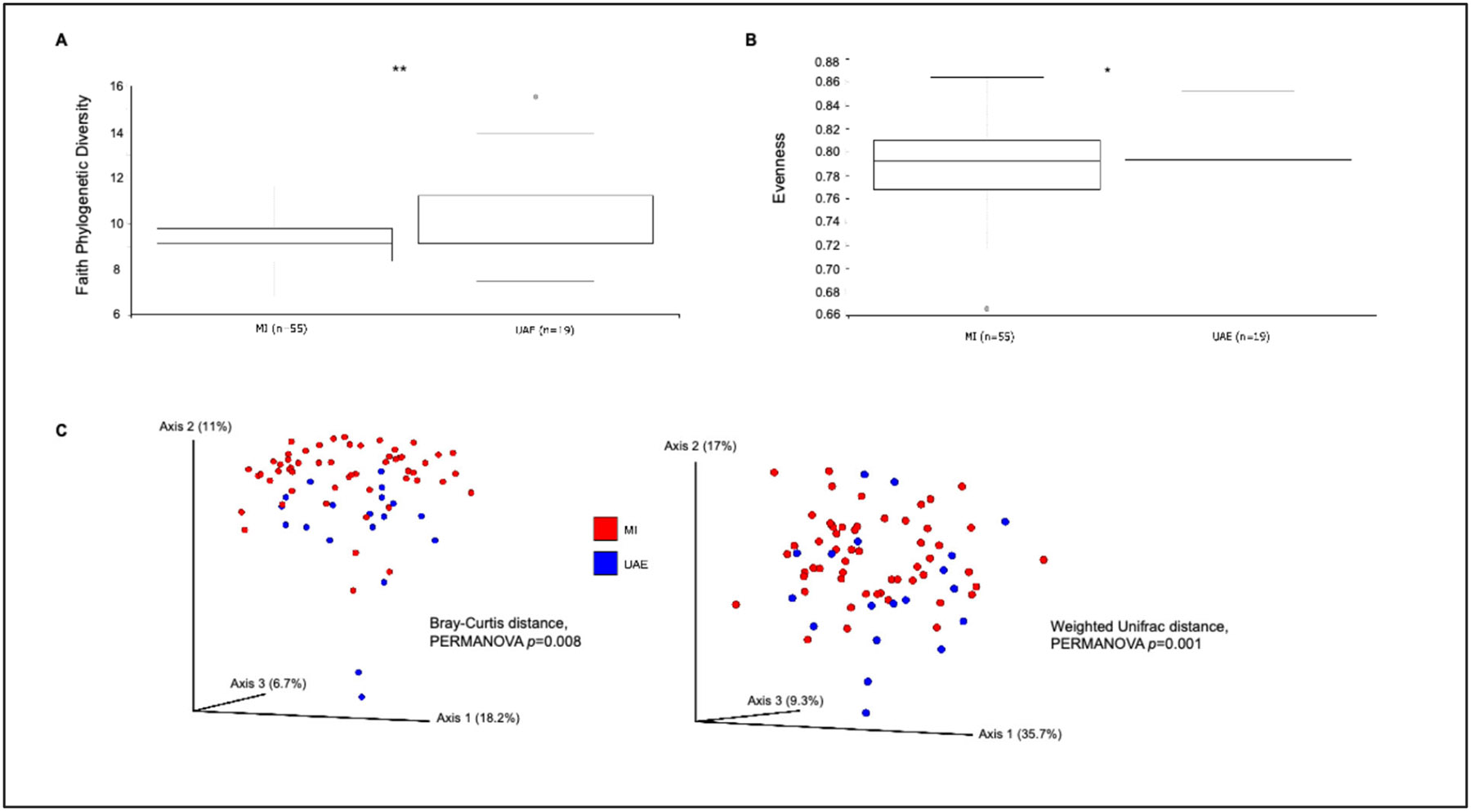
(**A**,**B**) Sputum bacterial alpha-diversity between study sites (Michigan (*n* = 55) and United Arab Emirates, U.A.E. (*n* = 19) * *p* ≤ 0.05; ** *p* ≤ 0.001. (**C**) Principal coordinates analysis based on beta-diversity measures (Bray–Curtis distance, *p* = 0.008; Unifrac distances, *p* = 0.001) showing variation in overall sputum bacterial composition associated with study site.

**Figure 2.**
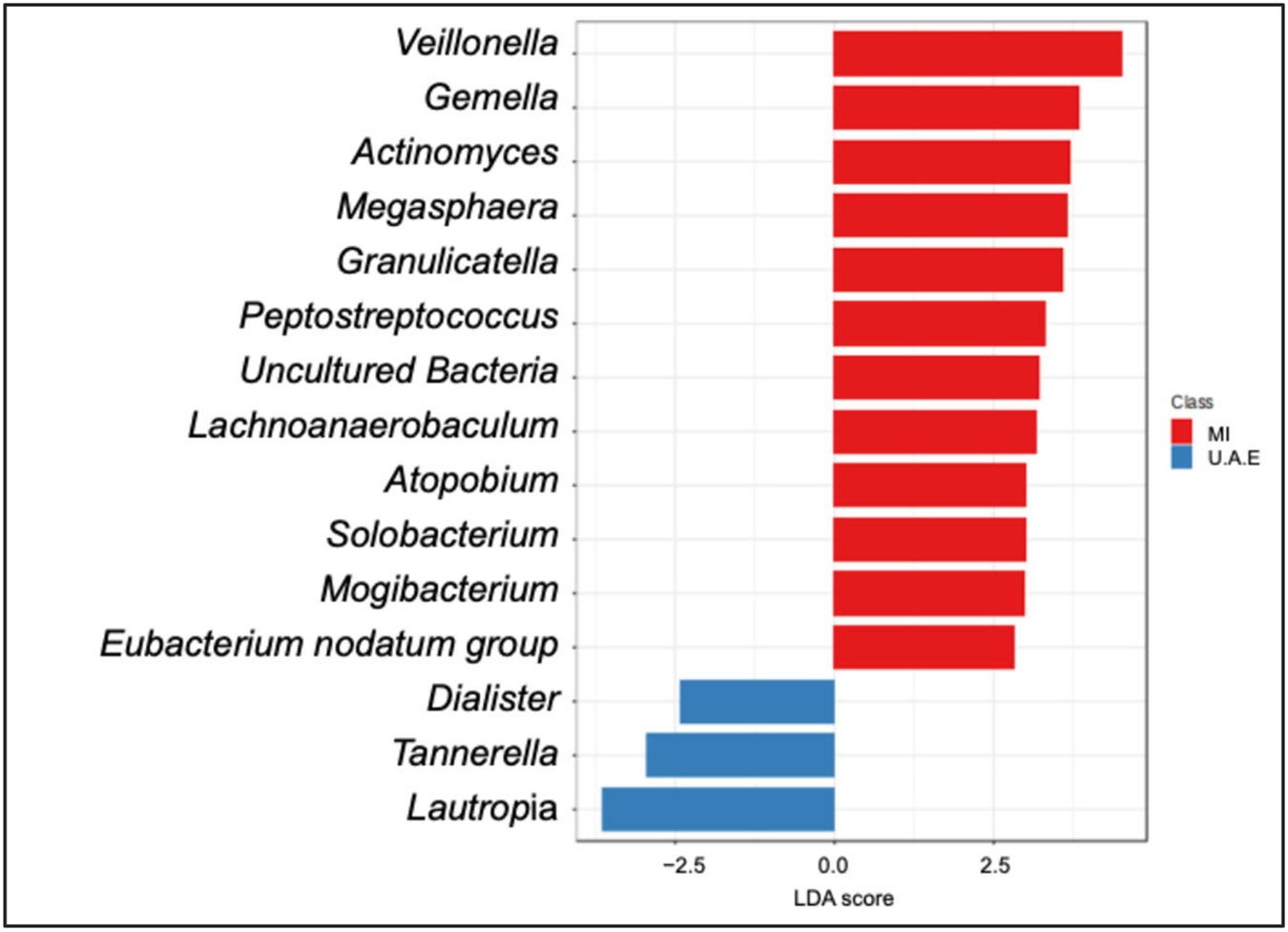
Differentially abundant bacterial genera between the study sites by LefSe analysis. LDA score > ∣2.5∣, padj ≤ 0.05). Positive LDA score values indicate enrichment in the MI group. Negative LDA scores conversely indicate enrichment in the UAE group.

We first examined and compared sputum bacterial community composition across all samples in both cohorts. Alpha-diversity analysis revealed significantly higher phylogenetic diversity (*p* = 0.001, Faith PD), but lower species evenness (*p* = 0.03) in the U.A.E. cohort (Figure 1A,B). Beta-diversity analyses based on Bray–Curtis or Unifrac distances also demonstrated that study site was associated with overall variation in sputum bacterial community composition, as the samples appeared to stratify largely along axes 2 and 3 (which, respectively, explained 11% and 6.7% of the variation in the data by Bray–Curtis distance, and 17% and 9.3% by weighted Unifrac distance) (Figure 1C). Linear Discriminant Analysis Effect Size (LEfSe) identified several bacterial genera associated with study site (Figure 2, LDA score > ∣2.5∣, padj ≤ 0.05). This is a two-step statistical methodology for metagenomic microbial biomarker analysis as described [37] and applied in other studies [39-41]. The MI cohort was enriched in *Veillonella*, *Gemella*, and *Actinomyces*, among others, while the U.A.E. cohort was enriched in *Lautropia*, *Tannerella*, and *Dialister*. Data plots from LEfSe conventionally show the LDA score, which represents the strength of each taxon in differentiating the two groups of interest.

### 3.2. Sputum Bacterial Community Composition and Asthma Status Across Both Cohorts

**Figure 3.**
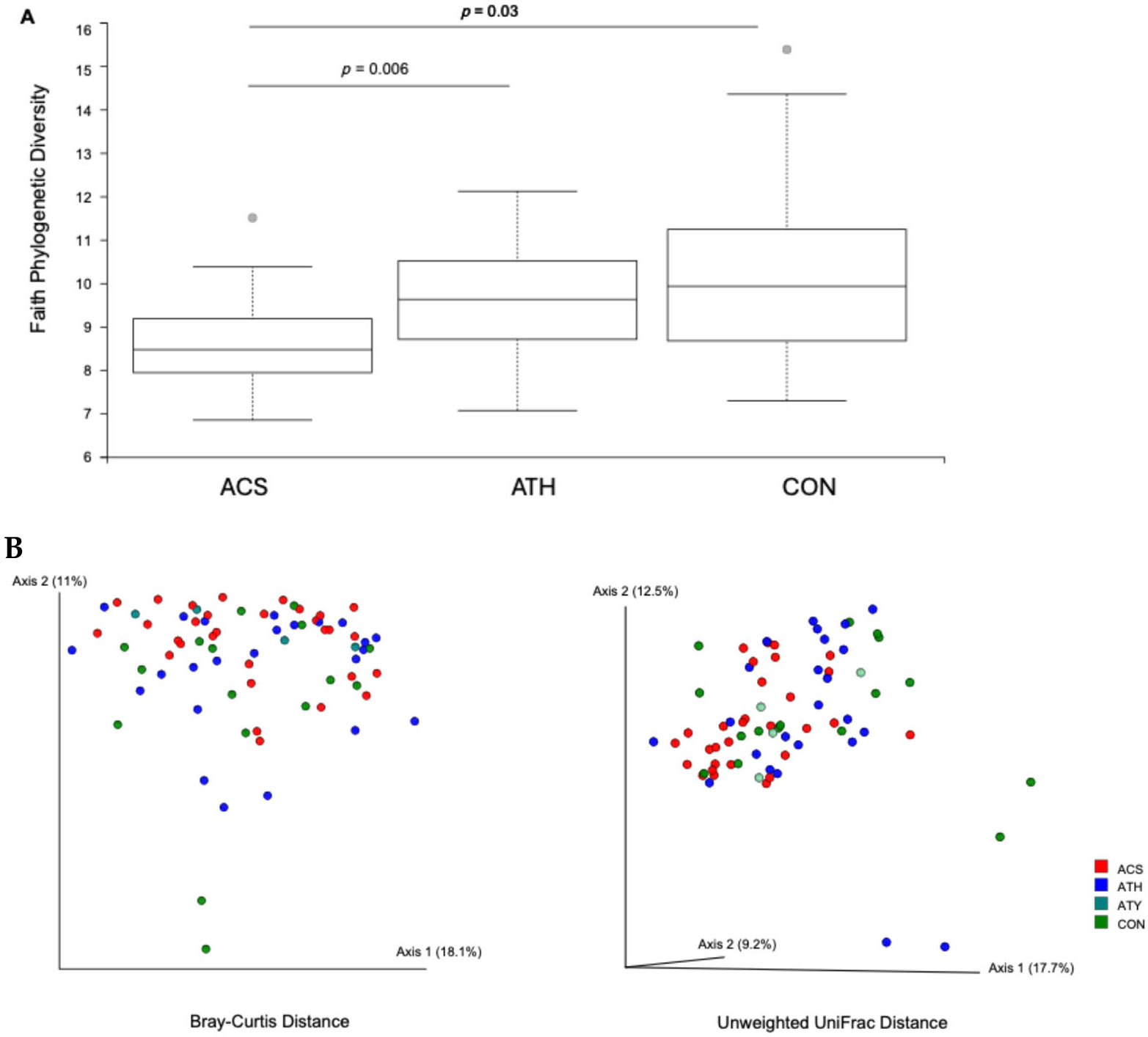
(**A**) Sputum bacterial phylogenetic diversity is lowest in the asthmatic group on inhaled corticosteroid treatment (ACS, *n* = 30), compared to those not on inhaled steroids (ATH, *n* = 24, *p* = 0.006) or healthy controls (CON, *n* = 20, *p* = 0.03). (**B**) Principal coordinates analysis by Bray–Curtis (left) or unweighted Unifrac (right) distance, showing the variation in overall sputum bacterial composition, largely driven by the ACS group (*p* = 0.008). Bray–Curtis distance PERMANOVA, (*p* = 0.008), unweighted Unifrac distance PERMANOVA, (*p* = 0.013). ATY, atopic non-asthma control (MI cohort only).

Using the data from both cohorts, we next examined if sputum bacterial composition, by alpha- or beta-diversity measures, differed in relation to asthma status and whether or not asthmatic patients were on inhaled corticosteroid (ICS) treatment. The latter is of particular interest because prior studies have shown that use of ICS is associated with alterations in the airway microbiome in asthmatic adults [4,9,10]. Phylogenetic diversity differed across the clinical groups (Kruskal–Wallis *p* = 0.03), being lowest in the asthmatic group on inhaled corticosteroid treatment (ACS). Faith PD differed most significantly between the ACS group and those not on ICS therapy (ATH) (*p* = 0.006, q = 0.03), followed by between the ACS and healthy control groups (*p* = 0.03, q = 0.09) (Figure 3A). Beta-diversity analyses, by both Bray–Curtis and Unifrac distance measures, also showed that variation in bacterial composition between sputum samples was significantly associated with ICS treatment (Figure 3B). Thus, asthma-associated differences were discerned in spite of the overall variation by study site.

We next performed analyses focused on asthmatic subject data only. Differences in bacterial alpha- and beta-diversity by study site were again noted (*p* < 0.05), with U.A.E. asthma patients exhibiting lower species evenness (*p* = 0.002) and Shannon diversity (*p* = 0.05). Bacterial genera that differed between the two sites by edgeR analysis are shown in Table 2.

Nonetheless, despite these site-associated differences, we again observed significantly lower phylogenetic diversity in ACS compared to ATH subjects (*p* = 0.013) among asthmatic subjects from both cohorts. This was reflected in decreased relative abundance in the ACS group of the following bacterial genera: *Peptococcus*, *Eubacterium brachy* group, *Selenomonas*, and *Alloprevotella* (LeFSe *p* ≤ 0.05). We also conducted a differential abundance analysis controlling for study site, using a zero-inflated binomial model implemented in MaAsLin2 [42]. Even after controlling for study site, *Alloprevotella*, *Treponema*, *Tannerella*, *Eubacterium brachy* group, and *Eubacterium nodatum* group still demonstrated lower relative abundance in the ACS group (FDR ≤ 0.05) (Table 3).

### 3.3. Sputum Bacterial Community Composition and Obesity-Associated Asthma

**Figure 4.**
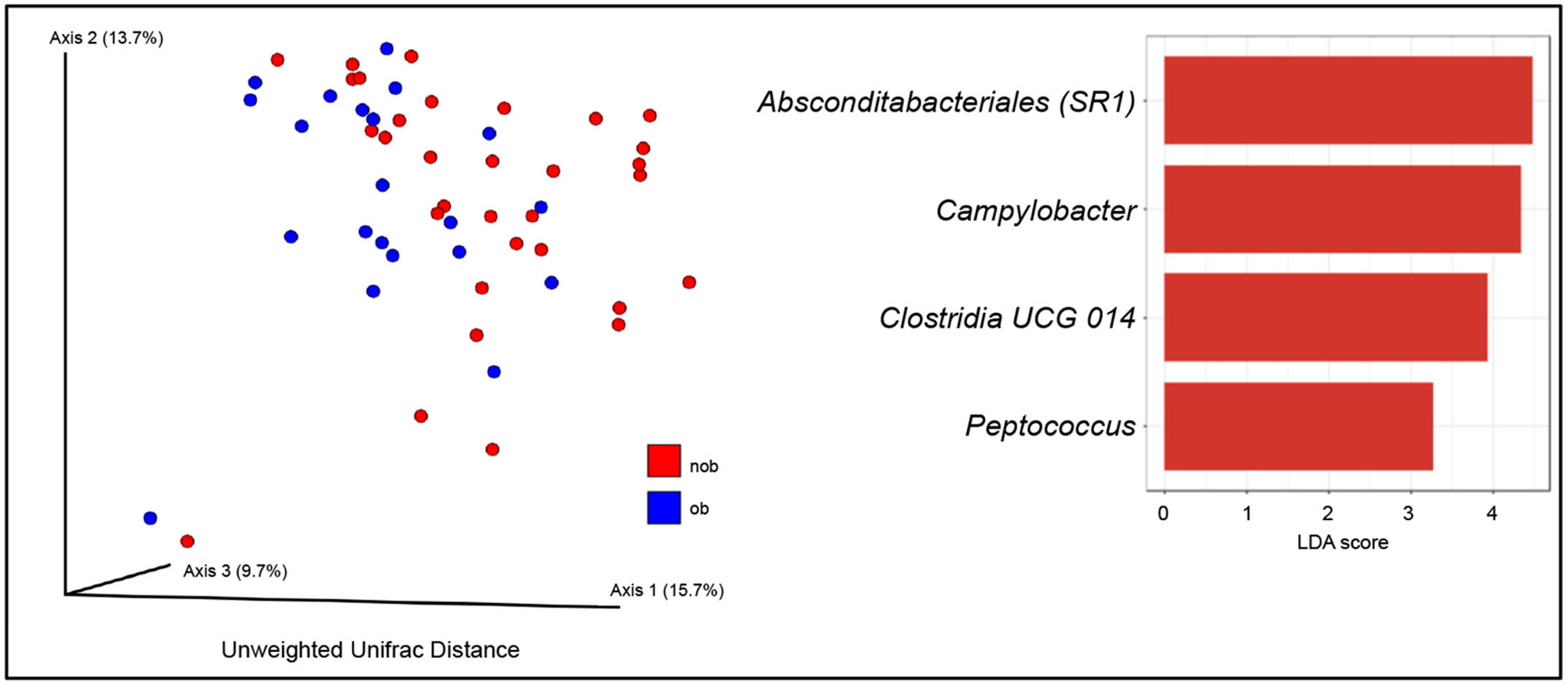
Principal coordinates analysis using unweighted Unifrac distance showing airway bacterial composition difference between obese (ob) and non-obese (nob) asthmatic subjects (PERMANOVA *p* = 0.004) across both cohorts. Differentially abundant bacterial genera between nob and ob asthma identified by LefSe (LDA score > ∣3.0∣, FDR < 0.05). Positive LDA scores indicate enrichment in the nob group.

We also explored whether obesity was associated with differences in airway bacterial composition, as this has been reported previously [8,43]. Principal coordinates analysis (PCoA) using Bray–Curtis distances demonstrated an association that was borderline significant among all participants and also among asthma subjects only (distance-based PERMANOVA *p* = 0.06). However, PCoA analysis by unweighted Unifrac distance, a phylogeny-based distance measure, revealed a statistically significant difference between non-obese (BMI ≤ 30) and obese (BMI ≥ 30) subjects with asthma (*p* = 0.004) (Figure 4). When weighted Unifrac was used, the association was not significant, which suggested that less prevalent taxa contribute to the observed differences between non-obese and obese subjects with asthma. LeFSe analysis identified 4 differentially abundant genera: *Absconditabacteriales_SR1*, *Campylobacter*, *Clostridia UCG 014*, and *Peptococcus*, all more abundant in the non-obese asthma group (LDA score > 3.0, FDR *p* < 0.05). Conversely, bacteria enriched in the obese asthma group included *Rothia*, *Gemella*, *Streptococcus*, *Actinobacillus*, and *Prevotella* (LefSe *p* < 0.05). *Absconditabacteriales_SR1* continued to meet FDR threshold for significance after controlling for study site.

### 3.4. Airway Microbiota Associations with Asthma Clinical Factors in the U.A.E. Cohort

**Figure 5.**
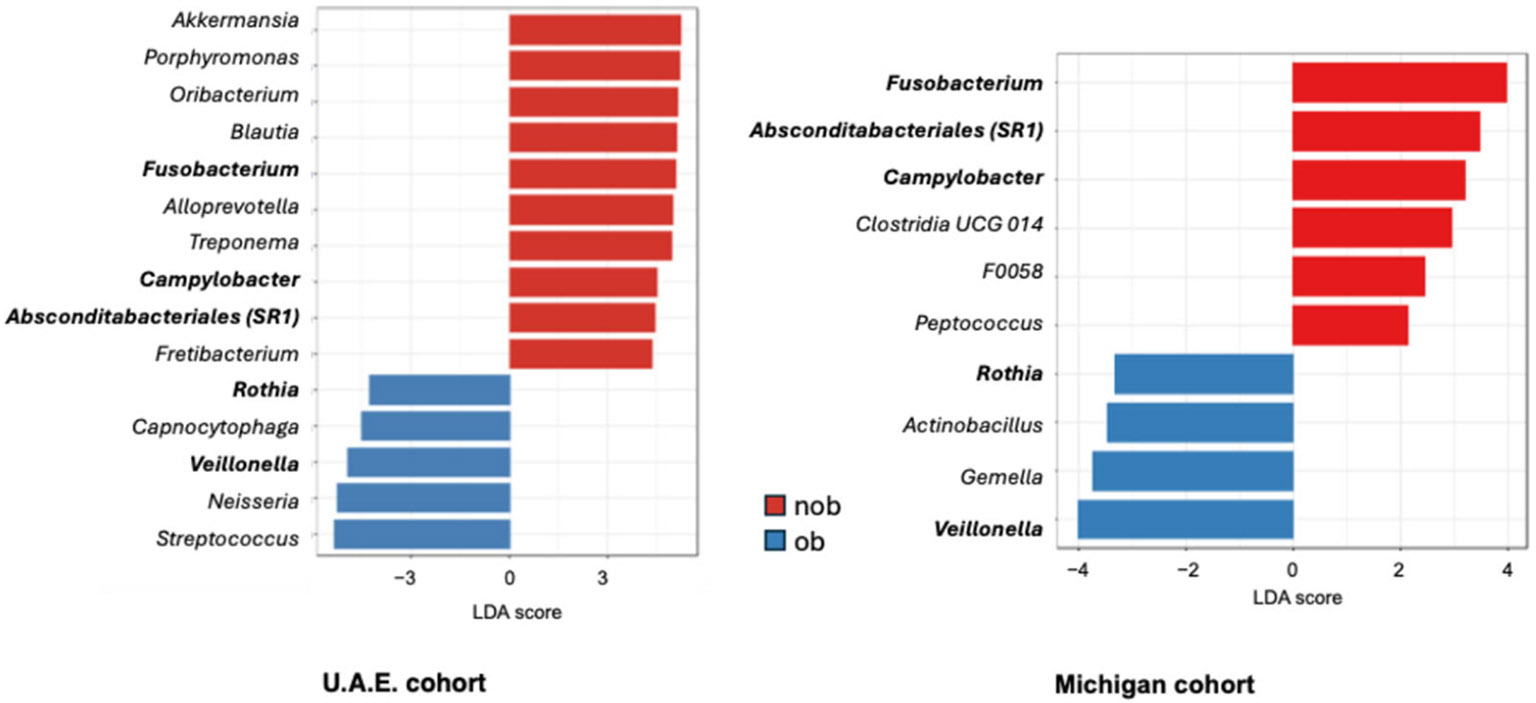
LefSe analysis of U.A.E. sputum microbiota data only demonstrating bacterial genera differentially abundant between obese (ob) and non-obese (nob) participants. Genera in bold text indicate those associated with nob and ob status in both cohorts.

**Figure A1.**
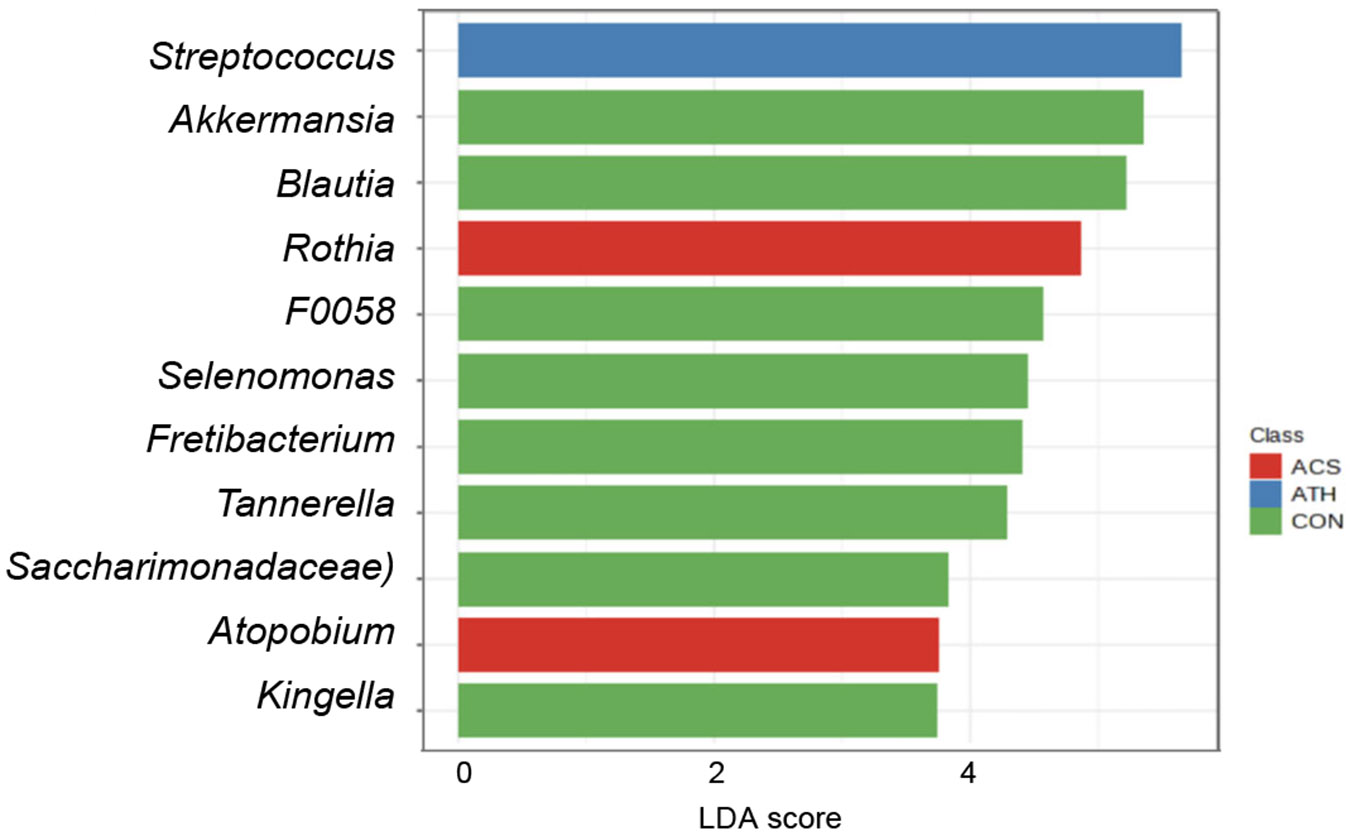
LefSe analysis of U.A.E. sputum bacterial microbiota data showing bacterial genera identified as differentially abundant between asthmatic patients on inhaled corticosteroid treatments (ACS), those not on inhaled steroids (ATH), and healthy controls. (LDA score > 2.0, *p* < 0.05).

Given our findings from the combined cohort data, we lastly examined if similar clinical associations (i.e., ICS treatment and obesity status) were present in the U.A.E. data alone. We pursued this since the U.A.E cohort was a much smaller sample size and reasoned that observance of similar clinical associations would lend support to conserved asthma-related microbiota differences across geographic regions. Indeed, these clinical factors demonstrated microbial associations even within the U.A.E. cohort. LeFSe analysis identified several differentially abundant bacterial genera between ACS, ATH, and control groups among the U.A.E. subjects (LDA score > 2.0, *p* < 0.05). These included higher relative abundances of *Rothia* and *Atopobium* in the ACS group and *Streptococcus* in the ATH group (Figure A1). Among the several bacterial genera enriched in the control group was *Selenomonas*, which we noted was also strongly associated with healthy controls in the Michigan cohort (LDA score = 4.0 in both MI and U.A.E. data). We also identified differentially abundant genera between obese and non-obese participants in the U.A.E. cohort (Figure 5). Notably, several of these genera were also associated with obese status in the Michigan cohort data. These similarly associated bacterial genera included *Fusobacterium*, *Campylobacter*, and *Absconditabacterales*, which were enriched in non-obese subjects in both cohorts, while *Rothia* and *Veillonella* similarly associated with obese status in both cohorts (Figure 5).

## 4. Discussion

This is the first study to explore whether the composition of airway bacterial microbiota differs between adults with or without asthma residing in the U.S. and U.A.E., which represent contrasting climate regions but have high asthma burden in common [21,22,44-47]. Existing evidence has reported asthma-associated differences in the airway microbiome from region-specific studies and cohorts [3,9,10,20,48-50], yet comparative analyses using merged data from different cohorts has rarely been pursued. Our re-processing of existing 16S rRNA gene sequence data, generated using the same platform and sequencing approach, allowed us to analyze a merged dataset and identify asthma-related clinical factors shared across both cohorts that were associated with differences in airway microbiota composition.

An important strength of this study is the re-processing of existing 16S rRNA gene sequencing data to generate a merged bacterial profile (taxonomic) dataset from both the Michigan and U.A.E. cohorts. This has rarely been pursued in studies of the airway microbiome in adults with chronic airway disease, much less in chronic asthma. While there are inherent challenges to merging independently generated data, attempts to do so are broadly important for the field of microbiome research in lung disease [51]. It is known that microbiota composition can vary greatly between individuals, and specific organisms of identified interest (e.g., associated with disease status or a clinical marker) can be incongruent between different studies of the same disease. Hence, we pursued this exploratory study in part as a demonstration of addressing this need. In the process, we examined for differences and commonalities of airway microbiome features associated with asthma clinical features, like inhaled corticosteroid use and obesity, that have previously been reported in other studies [4,5,9,10,43].

Analysis of the combined cohort data identified significant differences in sputum bacterial composition between the study sites by both alpha- and beta-diversity measures. We anticipated this may be the case, given geographic differences between our study sites as well as in study designs (e.g., recruitment/enrollment, sample collections), which are acknowledged limitations. We note, however, that the names of specific bacteria that differed in relative abundance between the cohorts may be less important from a functional implication standpoint. This is because 16S rRNA-based data do not provide precise information on potential gene functions or expressed pathways that bacteria possess, some of which will overlap across different but related species (e.g., those within the same genera). Functional redundancy among airway microbiota is largely unknown, and thus the specific differences in named organisms between the cohorts is of unclear biological significance at this time. We also acknowledge a need for larger sample sizes as a limitation of this study. This is a common challenge facing secondary analyses of pre-existing microbiome sequence data, especially when attempting to combine datasets across geographies or ethnicities. Our hope is that this study will provide an example that motivates further meta-analyses in the future.

Despite the site-associated variation in sputum bacterial composition, we nonethe-less discerned bacterial community differences associated with asthmatic status, particularly with ICS use, as well as with obesity status, an important comorbidity. These observations are broadly concordant with previously reported findings from other cohorts [4,5,8-10,43,48]. We discerned similar associations with ICS use and obesity status within the U.A.E. cohort data despite its smaller sample size. Overall, this suggests that the potential impact of inhaled corticosteroid treatment and obesity on the airway microbiome may be more universal, as also suggested by other cohort studies [9,10,43]. Notably, several bacterial genera differentially associated with obesity status shared similarities between the cohorts (Figure 5), such as enrichment in *Rothia* and *Veillonella* in obese participants and enrichment in *Fusobacterium* and *Campylobacter* in non-obese participants.

Overall, we believe findings from this exploratory study should inspire future studies of the lower airway microbiome involving larger numbers of patients from diverse regions, demographically and geographically. This diversity is crucial, as environmental exposures and lifestyle/cultural factors likely impact the microbiome’s structure and function. By characterizing the microbiome across geographically diverse cohorts, we may uncover unique signatures associated with these factors that could influence asthma progression and outcomes. Broadening our knowledge will pave the way for advances in precision medicine, as we will gain a better understanding of how disease phenotypes can shape or be shaped by the microbiota and the resulting implications for intervening or treating chronic airway diseases, such as asthma. Furthermore, moving beyond taxonomic-based analyses of microbiota composition, such as the use of metagenomic or meta-transcriptomic methods, will be necessary to offer more functional insights into airway microbiota-derived pathways and metabolic activities. These could reveal common themes across different populations on airway microbial functions that have clinical relevance.

## Figures and Tables

**Table 1.** Characteristics of adults with or without mild-to-moderate asthma included in the study.

Site	Status	Age	Sex (% Female)	Race (% White)	Body Mass Index (BMI)	Asthma Control Test Score	Use of Inhaled Corticosteroids
Michigan	Asthmatic (*n* = 44)	38	55%	75%	28	21	59%
	Healthy (*n* = 11)	46	64%	91%	25	not applicable	not applicable
U.A.E	Asthmatic (*n* = 10)	64	64%	0%	31	18	40%
	Healthy (*n* = 10)	41	78%	0%	24	not applicable	not applicable

Values are median or %; White race refers to an individual with predominantly European ancestry.

**Table 2.** Asthmatic subjects only. Differentially abundant sputum bacteria between MI and U.A.E. patients (edgeR analysis). Positive log 2FC value indicates enrichment in U.A.E. samples.

Organism	Log 2FC	FDR-Adjusted *p*
*Prevotella nigrescens*	2.32	≤0.001
*Prevotella histicola*	2.9	≤0.001
*Prevotella oulorum*	1.9	0.013
*Solobacterium moorei*	−2.2	0.013
*Prevotella salivae*	1.4	0.029
*Prevotella denticola*	1.8	0.033
*Rothia mucilaginosa*	1.9	0.037
*Prevotella melaninogenica*	1.2	0.037
*Uncultured Bacteroidetes*	−1.8	0.037

**Table 3.** Differentially abundant sputum bacterial genera between asthmatic subjects on (ACS) vs. those not on (ATH) inhaled steroid treatment, after controlling for study site. Positive log 2FC values indicate higher relative abundance in ATH group and, conversely, lower in the ACS group.

Organism	Log 2FC	FDR-Adjusted *p*
*Alloprevotella*	0.7	0.01
*F0058*	2.2	0.02
*Treponema*	1.2	0.03
*Tannerella*	0.8	0.03
*Eubacterium brachy* group	1.1	0.05
*Eubacterium nodatum* group	0.6	0.05

## Data Availability

The originally generated 16S rRNA gene sequence files from the Michigan study cohort are deposited under NCBI BioProjectID PRJNA899889 [8]. Specific sample data used in this study can be provided upon request. Data from the U.A.E. cohort are available upon request to the authors, as these data are not publicly available due to privacy or ethical restrictions.

## Abbreviations

The following abbreviations are used in this manuscript:

ACS: asthma on inhaled corticosteroid treatment
ATH: asthma not on inhaled corticosteroid treatment
Faith PD: Faith phylogenetic diversity index
ICS: inhaled corticosteroid
LEfSe: linear discriminant analysis effect size
MaAsLin2: Microbiome Multivariate Association with Linear Models
MI: Michigan
nob: non-obese
ob: obese
QIIME2: Quantitative Insights Into Microbial Ecology
U.A.E.: United Arab Emirates
